# Contribution of Neuropilin-1 in Radiation-Survived Subclones of NSCLC Cell Line H1299

**DOI:** 10.3390/cimb43030085

**Published:** 2021-09-22

**Authors:** Kaori Tsutsumi, Ayaka Chiba, Yuta Tadaki, Shima Minaki, Takahito Ooshima, Haruka Takahashi

**Affiliations:** 1 Faculty of Health Sciences, Hokkaido University, Sapporo 060-0812, Japan; 2 Division of Radiology and Nuclear Medicine, Sapporo Medical University Hospital, Sapporo 060-8556, Japan; chiba-aya@sapmed.ac.jp; 3 Department of Radiological Technology, Saiseikai Otaru Hospital, Otaru 047-0008, Japan; scgftsjbq@gmail.com; 4 Department of Radiological Technology, Sapporo Spine Clinic, Sapporo 060-0042, Japan; nekobus7031@gmail.com; 5 Department of Radiological Technology, Tomakomai City Hospital, Tomakomai 053-8567, Japan; t.ooshima@tomakomai-city-hospital.com; 6 Department of X-ray Technology, Sapporo City General Hospital, Sapporo 060-8604, Japan; takahashi-haruka@city.sapporo.jp

**Keywords:** non-small cell lung cancer, radiotherapy, repopulated tumor, neuropilin, motility

## Abstract

Non-small cell lung cancer (NSCLC) is an aggressive lung cancer accounting for approximately 85% of all lung cancer patients. For the patients with Stages IIIA, IIIB, and IIIC, the 5-year survival is low though with the combination with radiotherapy and chemotherapy. In addition, the occurrence of tumor cells (repopulated tumors) that survive irradiation remains a challenge. In our previous report, we subcloned the radiation-surviving tumor cells (IR cells) using the human NSCLC cell line, H1299, and found that the expression of neuropilin-1 (*NRP*-1) was upregulated in IR cells by the microarray analysis. Here, we investigated the contribution of neuropilin-1 to changes in the characteristics of IR cells. Although there were no differences in angiogenic activity in the tube formation assay between parental and IR cells, the cell motility was increased in IR cells compared to parental cells in the cell migration assay. This enhanced cell motility was suppressed by pretreatment with anti-NRP-1 antibody. Although further studies are necessary to identify other molecules associated with NRP-1, the increase in cellular motility in IR cells might be due to the contribution of NRP-1. Inhibition of NRP-1 would help control tumor malignancy in radiation-surviving NSCLC.

## 1. Introduction

Non-small cell lung cancer (NSCLC) is an aggressive lung cancer and one of the most common causes of death in Japan and other countries worldwide, accounting for approximately 85% of all lung cancer patients [1,2,3]. The standard therapy for the localized small NSCLC (Stage I) is surgery with a high survival rate and for inoperable Stage I patients, stereotactic ablative radiotherapy also lead a good overall survival [4]. However, despite the recent development of radiotherapy techniques and radiotherapy combined with chemotherapy, the 5-year overall survival rate for patients with Stages IIIA, IIIB, and IIIC is only 36%, 26%, and 13%, respectively [1,5,6]. Furthermore, in radiation therapy, the occurrence of tumor cells that survive radiation exposure remains a challenge and the characteristics of repopulated tumors remain unclear [7].

In our previous study, we established the model-cells of the repopulated tumor cells after radiotherapy (IR cells) using a human NSCLC cell line (H1299) and reported an upregulated and downregulated gene using comprehensive microarray analysis, and also found increased cellular motility and invasiveness before irradiation [8,9]. Although there have been many alterations in gene expression by comprehensive analysis, the function of many of these genes in IR cells remains unclear. In the present study, we focused on neuropilin-1 (*NRP-1*) among the upregulated genes in the IR cells that survived 10 Gy of X-ray irradiation, that was 2.4-fold upregulated than the original tumor cells in microarray analysis [8].

Neuropilin (NRP) is a transmembrane protein found specifically in vertebrates. There are two types of NRP, NRP-1 and NRP-2, and their expression has been reported in various cells, such as endothelial cells, neurons, islet cells, hepatocytes, melanocytes, and osteoblasts [10,11]. In malignant tumors, the expression of *NRP* has also been confirmed in lung, pancreatic, prostate, breast, and ovarian cancer cells [11,12,13,14,15], and NRP-1 plays a mediator in tumor development associated with tumor initiation, tumor growth, and angiogenesis [10,11,16]. NRP-1 is also associated with cell migration-accompanying semaphoring 3F (SEMA3F), in lung cancer cell line [17] or modulation of transforming growth factor-β1 (TGF-β1)-induced epithelial-mesenchymal transition in NSCLC [18].

In the present study, we investigated the contribution of NRP-1 on tumor motility and angiogenesis in a repopulated tumor that survived 10 Gy of X-ray irradiation using H1299-IR cells.

## 2. Materials and Methods

### 2.1. Cells Culture and Reagents

The human NSCLC cell line, H1299, obtained from American Type Culture Collection (ATCC, Manassas, VA, USA), was maintained in Dulbecco’s modified Eagle’s medium (DMEM, Sigma, St. Louis, MO, USA) supplemented with 10% fetal bovine serum (FBS, Nichirei Biosciences Inc., Tokyo, Japan) at 37 °C in a humidified atmosphere of 95% air and 5% CO_2_. H1299-IR cells were previously exposed to 10 Gy of X-ray irradiation [8]. Human umbilical vein endothelial cells (HUVECs) were obtained from Lonza group Ltd. (Basel, Switzerland) and maintained in an Endothelial Cell Growth Medium-2 (EGM-2) Bullet Kit (Lonza Group Ltd., Basel, Switzerland) at 37 °C in a humidified atmosphere of 95% air and 5% CO_2_.

### 2.2. Reverse Transcription PCR (RT-PCR)

Total RNA was isolated from cells using the RNeasy Mini Kit (QIAGEN, Hilden, Germany) according to the manufacturer’s instructions. First-strand cDNA was synthesized using SuperScript III reverse transcriptase (Invitrogen, Carlsbad, CA, USA) and amplified by polymerase chain reaction for 30 cycles. Sequences of the oligonucleotide primer sets used for reverse transcription-PCR analysis were as follows: *NRP-1* forward primer, 5′-AGG ACAGAGACTGCAAGTATGAC-3′; *NRP-1* reverse primer, 5′-AACATTCAGGACCTCTCTTGA-3′; *gapdh* forward primer, 5′-CAACAGCCTCAAGATCATCA-3′, *gapdh* reverse primer, 5′-TTGACAAAGTGGTCGTTGAG-3′. The amplified DNA fragments were loaded onto a 1.5% agarose gel and visualized using the ChemiDoc Touch Gel Imaging System (Bio-Rad Laboratories, Inc., Hercules, CA, USA).

### 2.3. Tube Formation Assay

A total of 50,000 HUVEC cells were seeded on Matrigel containing 56% laminin, 31% collagen IV, and 8% entactin (BD Matrigel Matrix Growth Factor Reduced, Becton, Dickinson and Company, Franklin Lakes, NJ, USA) in a 12-well plate with the conditioned medium from H1299 and H1299-IR cells for 4 h at 37 °C and a 5% CO_2_ atmosphere. Tube formation was monitored using an IX71 microscope (Olympus Corporation, Tokyo, Japan), and the length of the elongated tube formation was measured using ImageJ (National Institutes of Health, Bethesda, MD, USA).

### 2.4. Enzyme-Linked Immunosorbent Assay (ELISA) for Vascular Endothelial Growth Factor (VEGF)

VEGF secretion in a conditioned medium of cell culture was detected by ELISA (VEGF human ELISA kit, Invitrogen) according to the manufacturer’s instructions. Absorbance at 450 nm was measured using a microplate reader (Bio-Rad model 680, Bio-Rad Laboratories, Inc., Hercules, CA, USA).

### 2.5. Cell Motility Assay

Cell motility was monitored with or without the introduction of the *NRP-1* expression and with or without treatment with NRP-1. To overexpress *NRP-1*, *NRP-1* the expression plasmids were introduced into the cells using FuGene HD (Roche Corp., Basel, Switzerland). After 24 h, cells were treated with anti-NRP-1 antibody for 24 h, trypsinized, and seeded onto a Transwell filter (Corning Inc., Corning, NY, USA) at a density of 3000 cells/well. After 16 h, the total number of cells that migrated to the lower surface of the filter was counted after staining with 0.1% crystal violet. The *NRP-1* expression plasmid was kindly provided by Dr. Seiji Takashima (Osaka University, Japan).

### 2.6. Immunopreciptation and Western Blotting

*NRP-1* expression plasmids were introduced by FuGENE HD (Roche Corp., Basel, Switzerland). After 24 h, the cells were lysed in a lysis buffer containing 50 mM Tris-HCl (pH 7.4), 150 mM NaCl, 5 mM MgCl_2_, 1% NP-40, 0.1% SDS, 0.5% sodium deoxycholate, 1 mM Na_3_VO_4_, and complete protease inhibitor (Roche, Indianapolis, IN, USA), and the supernatants were clarified by microcentrifugation. Fifty micrograms of protein were immunoprecipitated with 1 μg of anti-NRP-1 antibody using Immunoprecipitation Kit-Dynabeads ProteinG (Invitrogen Corp., Waltham, MA, USA). SDS-PAGE was performed, and separated proteins were transferred to polyvinylidene difluoride membranes (Bio-Rad, Hercules, CA, USA). The membranes were incubated with primary antibodies specific for NRP-1 (1:200 dilution, Santa Cruz Biotechnology, Inc., Dallas, TX, USA), α5β1 integrin (1:1000 dilution; Cell Signaling Technology, Inc., MA, USA), and αvβ3 integrin (1:1000 dilution; Santa Cruz Biotechnology, Inc., Dallas, TX, USA), followed by peroxidase-labeled secondary antibodies. Signals were developed using ECL Western Blotting Detection Reagent (GE Healthcare, Little Chalfont, UK) and the luminescent signals were detected X-ray films.

### 2.7. DNA Microarray Analysis

The parental and IR cells were seeded onto 60-mm dishes and after for 24 h, total RNA was extracted from the cells using the QIAGEN RNeasy kit (Qiagen, Chatsworth, CA, USA). The extracted RNA was labeled and hybridized onto a human microarray chip that targeted approximately 30,000 human genes. The detected signals were examined by computer analysis (Agilent Technologies Japan, Ltd., Tokyo, Japan).

### 2.8. Statististical Analysis

All statistical analyses were performed using SPSS Statistics version 18 (IBM Corp., Armonk, NY, USA). Comparisons between the two groups were performed using the Mann–Whitney U test. Error bars represent standard deviation (SD) values. Statistical significance was set at *p* < 0.05.

## 3. Results

### 3.1. Expression of NRP-1 in H1299-IR Cells

**Figure 1:**
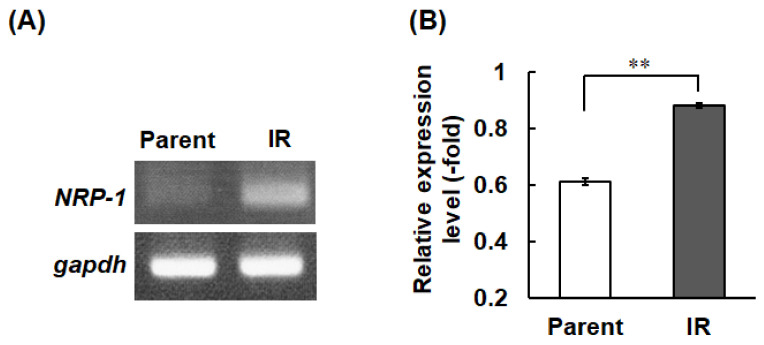
Expression levels of *NRP-1* mRNA. The amplified DNA images by 1.5% agarose gel electrophoresis after RT-PCR (**A**) and relative expression levels of *NRP-1* normalized to *gapdh* expression (**B**). Parent represents parental cells of H1299-IR cells (IR). The error bar indicates the standard deviation of the mean of pentaplicate samples. The double asterisks represent statistically significant values of *p* < 0.01.

To confirm the upregulation of *NRP-1* mRNA expression according to our previous report by microarray analysis [8], we first performed reverse transcription PCR (RT-PCR, Figure 1A) and compared the relative expression levels of glyceraldehyde-3-phosphate dehydrogenase (*gapdh*) mRNA between H1299-IR and parental cells, which are the original cells of the IR cells (Parent) (Figure 1B). The results show that the expression of *NRP-1* mRNA was 1.3-fold higher in IR cells than in the parental H1299 cells (Figure 1A,B).

### 3.2. Tube Formation of HUVECs

**Figure 2:**
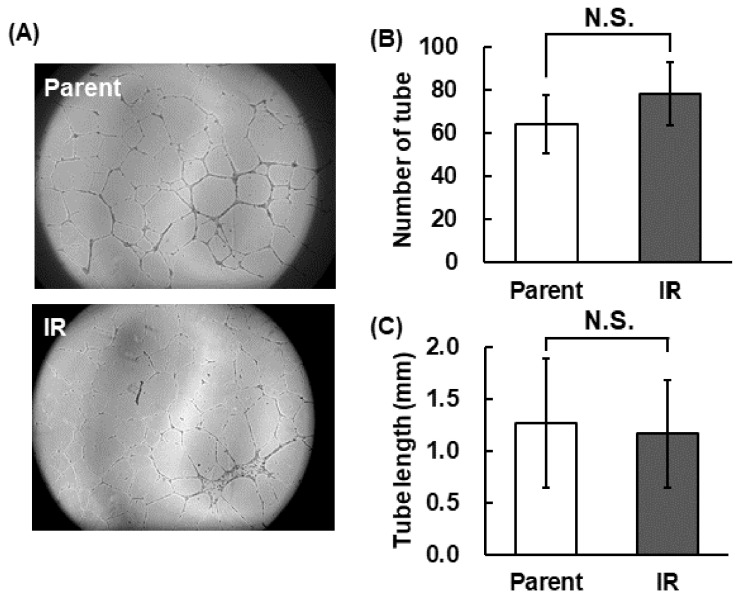
Angiogenic activity of parental H1299 and H1299-IR cells. (**A**) The images of tube formation. (**B**) The number of the formed tube. (**C**) The average length of the tube. The error bar indicates the standard deviation of the mean of quadruplicate samples. N.S. represents statistically “not significant”.

The angiogenic activity of parental H1299 (parent) and H1299-IR cells (IR) was estimated by a tube formation assay using HUVECs (Figure 2). The number of the formed tubes and the average length of the tube did not suggest differences between parental H1299 and H1299-IR cells (Figure 2A–C).

### 3.3. Secretion of VEGF

**Figure 3:**
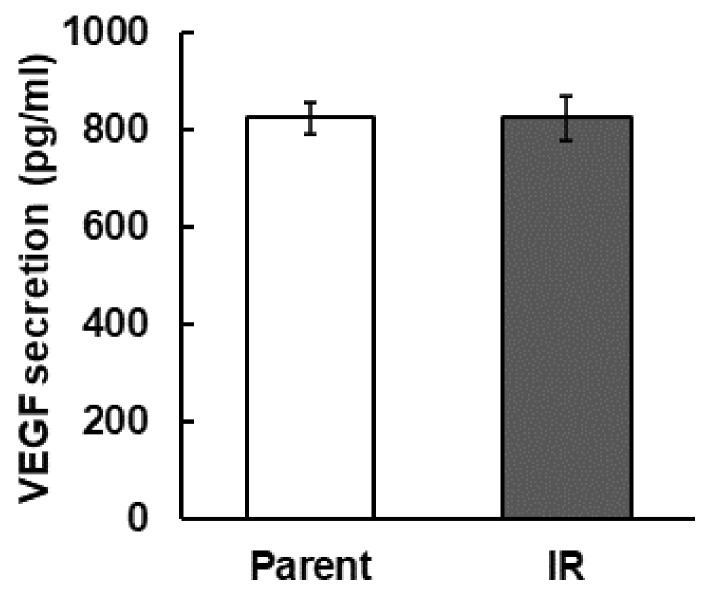
The secretion of VEGF was measured by ELISA assay.

Next, we compared the amount of VEGF secretion that promotes angiogenesis in tumors. However, we found no significant differences in these parameters (Figure 3).

### 3.4. Cell Motility of IR Cells

**Figure 4:**
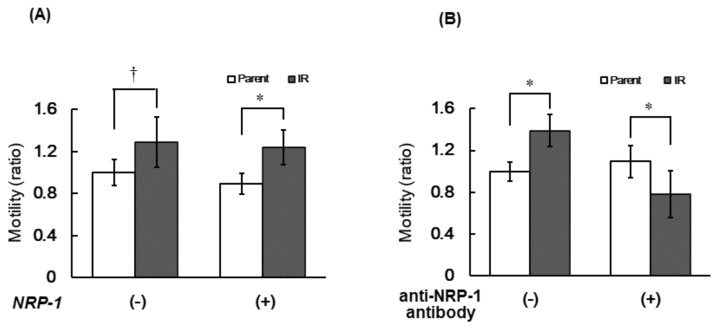
The cell motility was monitored by a Transwell migration assay with or without over expression of *NRP-1* (**A**) and with or without treatment with anti-NRP-1 antibody under over expression of *NRP-1* (**B**). A dagger (†) and an asterisk (*) indicate a statistically significant value of *p* < 0.1 and *p* < 0.05 respectively. The ratio of the motilities was normalized by the cell number in parental cells without *NRP-1* expression (**A**) and without treatment with anti-NRP-1 antibody (**B**).

To compare cell motility between parental and IR cells, we performed a cellular migration assay with or without the introduction of the *NRP-1* expression vector. IR cells showed no significant increase in cellular motility, but after the overexpression of *NRP-1*, the cells migrated well in the IR cells (approximately 1.3-fold, *p*-value = 0.02) compared with parental cells (Figure 4A). Furthermore, the increased motility in IR cells was inhibited by pretreatment with the anti-NRP-1 antibody (Figure 4B).

### 3.5. Interaction of NRP-1 with αVβ3 Integrins

**Figure 5:**
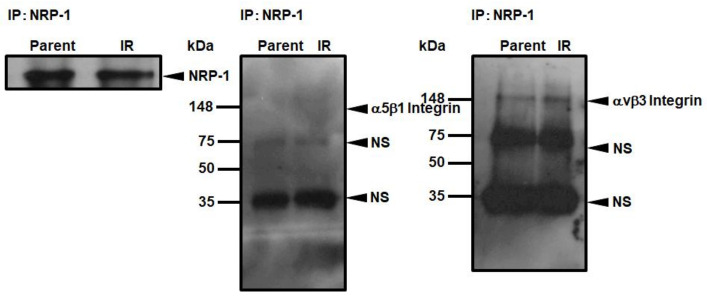
Interaction of NRP-1 with α5β1 and αVβ3 integrins. After overexpression of *NRP-1*, cell lysate was immunoprecipitated by anti-NRP-1, and these interactions were monitored by western blotting reacted with the proper antibodies. The arrows represent the specific and non-specific (NS) bands.

To confirm whether the increase in cellular motility in IR cells was due to the interaction of NRP-1 with the integrin family, we compared the interaction of α5β1 and αVβ3 integrins with NRP-1 after introducing the *NRP-1* expression vector in parental and IR cells (Figure 5). Despite the overexpression of *NRP-1*, no interaction was observed between NRP-1 and α5β1 integrin (Figure 5). While αVβ3 integrin interacted with NRP-1 in parental and IR cells, the interaction levels were the same (Figure 5).

### 3.6. Changes in NRP-1 Expression Profiles in Three Cell Lines Analyzed by Microarrays

The changes in gene expression were compared by microarray analysis between parental and IR cells in three cell lines (Table 1). Among 30,000 genes, *NRP-1* was commonly upregulated in IR cells compared with parental cells in three cell lines, H1299, A549 and MCF7 (Table 1).

## 4. Discussion

In the present study, we confirmed the upregulation of *NRP-1* mRNA expression in radiation-surviving NSCLC, H1299 (Figure 1), and found an increase in cellular motility in IR cells, especially in the cells that overexpressed *NRP-1* (Figure 4A). Moreover, these increased motilities were inhibited by the treatment with the anti-NRP-1 antibody (Figure 4B). However, no significant effect was observed in the upregulation of angiogenic activity in IR cells and no differences in VEGF secretion between parental and IR cells (Figure 2 and Figure 3).

In radiotherapy, suppressing the occurrence of radioresistant tumor cells and tumor repopulation are severe issues for tumor control [7], and studies to understand the cellular characteristics of repopulated tumors using model-cells have been vigorously performed recently [19,20,21,22,23]. These model-cells that survived irradiation commonly show increased malignancy with increased invasiveness, adhesion, and motility, as in clinical cases. In our previous study, we also suggested that the increased gelatinase activity due to the increased matrix metalloproteinase 1, 2 and 9 (*MMP1*, *MMP2* and *MMP9*) expression in IR cells was responsible for the increased invasive capacity of IR cells [9]. Ishihara et al. also reported that specific glycosphingolipids are highly expressed in invasive, irradiation-tolerant lung cancer cells [22], and the invasive ability depends on dephosphorylation of the myosin regulatory light chain [22]. On the other hand, as a key molecule for increased cell adhesion in IR cells, we have indicated the possibility of paxillin, vinculin, and phosphorylated focal adhesion kinase (FAK) [9]. However, the molecule responsible for the increased motility of IR cells has not yet been identified. In the present study, we showed the possibility that NRP-1 may be a key molecule in the enhancement of cell motility in radiation-surviving tumor cells. Interestingly, these increased motilities were suppressed by the pretreatment with the anti-NRP-1 antibody only in the cells that were overexpressed by the NRP-1 in IR cells. Furthermore, this suppression of the motility was below the basal level of the parental cells (Figure 4B). There is one possible explanation for why the cellular motility in *NRP-1* overexpressed IR cells was suppressed below the basal levels. Our previous study showed that 351 genes were upregulated and 368 genes were downregulated among 30,000 of human genes in IR cells [8]. Among them, there may be the molecules that interact with NRP-1 involving the cellular motility. Pretreatment with anti-NRP-1 antibody may suppress the activities of both endogenous and overexpressed molecules that interact with NRP-1. Although the present study did not find any integrin family that strongly interacts with NRP-1 in IR cells (Figure 5), further investigation on the changes in localization and inter-actions of NRP-1 with integrin family or with the other molecules are needed to further understand the molecular mechanisms that enhance cellular motility in IR cells.

A recent study demonstrated that NRP-1 modulates TGF-β1-induced epithelial-mesenchymal transition (EMT) in NSCLC and enhances cell migration and invasion, which are suppressed by the introduction of the short hairpin RNA of *NRP-1* into the cells [18]. Furthermore, radiation-induced EMT and glial-mesenchymal transition have been observed in NSCLC cell line (A549) and malignant glioma cell line (T98G and KMG4), respectively [24,25]. Although the epithelial mesenchymal transition after radiation in H1299 needs to be further investigated, there is also a possibility of the association of up-regulation of *NRP-1* in IR cells with the enhancement of the tumor migration and invasion caused by the radiation-derived EMT. The suppression of NRP-1 is effective in controlling the malignancy of NSCLC and might be more effective in repopulated tumors after radiotherapy.

NRP-1 is also a well-known VEGF receptor that regulates angiogenesis by controlling VEGF signaling [16]. In addition, in hypoxic tumor cells, upregulation of VEGF and bFGF by the hypoxia inducible factor 1 (HIF-1) signal transduction pathway and further upregulation of HIF-1 activity by irradiation lead the tumor radio resistance through vascular radioprotection [26,27,28,29]. Therefore, we hypothesized that up-regulation of *NRP-1* in IR cells may also affect to their enhancement of angiogenesis. However, IR cells did not affect VEGF secretion and its angiogenic activities (Figure 1, Figure 2 and Figure 3). Our present study showed that in IR cells, NRP-1 plays a role in enhancing tumor motility, but not angiogenesis.

Upregulation of *NRP-1* expression in IR cells was showed in three deferent cell lines by the comprehensive microarray analysis (Table 1). Further study is required to validate whether this upregulation of *NRP-1* is a common phenomenon among tumor cells that survive irradiation.

The limitations need to be acknowledged in present study. In the present study, we examined with the *p53*-null H1299 for NSCLC. Further investigation is needed using other cell lines of NSCLC. Next, the present experiment is a study using a cell line and is not a clinical validation.

In conclusion, the present study showed that upregulation of *NRP-1* led to increased tumor motility in radiation-surviving tumor cells, which could be suppressed by the treatment of anti-NRP-1 antibody to a greater extent than the tumor cells before irradiation. Although further studies are required to elucidate the molecules that interact with NRP-1 and to verify the effect of anti-NRP-1 antibody on repopulated tumors and in the improvement of tumor control after radiotherapy in NSCLC, NRP-1-targeted investigation on repopulated tumor after radiotherapy might open a road to lead the possibility of a tumor control that survived radiotherapy.

## Figures and Tables

**Table 1:** Upregulation of *NRP-1* expression in IR cells compared with parental cells.

Cell Line	Species	Tissue	Case History	Fold-Change
H1299	Homo sapiens	lung	non-small cell lung cancer canceradenocarcinoma	2.44
A549	Homo sapiens	lung	non-small cell lung cancer	1.35
MCF7	Homo sapiens	breast	breast cancer	2.35

## Data Availability

The data that support the findings of this study are not publicly available. However, data are available from the authors upon reasonable request.
